# Contribution of the private sector healthcare service providers to malaria diagnosis in a prevention of re-introduction setting

**DOI:** 10.1186/s12936-016-1570-5

**Published:** 2016-10-18

**Authors:** Sumadhya Deepika Fernando, Priyani Dharmawardana, Geethanee Epasinghe, Niroshana Senanayake, Chaturaka Rodrigo, Risintha Premaratne, Rajitha Wickremasinghe

**Affiliations:** 1 Department of Parasitology, Faculty of Medicine, University of Colombo, Colombo, Sri Lanka; 2 Anti Malaria Campaign, Ministry of Health, 555/5 Public Health Complex, Narahenpita, Colombo 5, Sri Lanka; 3 Department of Clinical Medicine, Faculty of Medicine, University of Colombo, Colombo, Sri Lanka; 4 Department of Public Health, Faculty of Medicine, University of Kelaniya, Kelaniya, Sri Lanka

## Abstract

**Background:**

Sri Lanka is currently in the prevention of re-introduction phase of malaria. The engagement of the private sector health care institutions in malaria surveillance is important. The purpose of the study was to determine the number of diagnostic tests carried out, the number of positive cases identified and the referral system for diagnosis in the private sector and to estimate the costs involved.

**Methods:**

This prospective study of private sector laboratories within the Colombo District of Sri Lanka was carried out over a 6-month period in 2015. The management of registered private sector laboratories was contacted individually and the purpose of the study was explained. A reporting format was developed and introduced for monthly reporting.

**Results:**

Forty-one laboratories were eligible to be included in the study and 28 participated by reporting data on a monthly basis. Excluding blood bank samples and routine testing for foreign employment, malaria diagnostic tests were carried out on 973 individuals during the 6-month period and nine malaria cases were identified. In 2015, a total of 36 malaria cases were reported from Sri Lanka. Of these, 24 (67 %) were diagnosed in the Colombo District and 50 % of them were diagnosed in private hospitals.

**Conclusions:**

An equal number of cases were diagnosed from the private sector and government sector in the Colombo District in 2015. The private sector being a major contributor in the detection of imported malaria cases in the country should be actively engaged in the national malaria surveillance system.

## Background

Approximately, 3.2 billion people still remain at risk of malaria globally. In 2015, there were an estimated 214 million cases of malaria and 438,000 deaths [1]. Many countries and regions worldwide have, in the past few years pledged to eliminate malaria, and this requires commitment from both government and private sectors [2].

Influenced by the sustained reduction in the morbidity and mortality of malaria since 1999, the anti-malaria campaign (AMC) of Sri Lanka embarked on phased elimination of malaria from the country by the end of 2014. The end of the 30-year-long civil conflict and a committed effort aided by funding from Global Fund to Fight AIDS, Tuberculosis and Malaria (GFATM) made it possible for Sri Lanka to interrupt indigenous malaria transmission by October 2012 [3]. Once malaria transmission is interrupted, the major challenge is the prevention of reintroduction through strengthening of surveillance and management of imported malaria cases [3]. Since October 2012, all malaria cases reported in Sri Lanka have been imported cases. Sri Lanka was certified malaria free on 5th September 2016.

Re-introduction of malaria remains a possibility as receptivity is high with a high abundance of malaria vectors, presence of a non-immune population and more importantly, a decreasing level of interest and awareness among clinicians, administrators and health care providers. Referrals for investigations for malaria parasites by health care providers are poor as malaria is rarely considered in the differential diagnoses of fever. This is despite the availability of diagnostic services (quality assured microscopy to identify and quantify malaria parasites and rapid diagnostic test kits (RDTs) to detect malaria antigens) throughout the country. There have been unacceptable delays, sometimes even up to 30 days, to diagnose malaria [4]. Fifteen cases of severe malaria were reported, some probably as a result of delayed diagnosis in 2013/2014. Despite this, zero malaria mortality among indigenous cases has been sustained in the country since 2007 [4]. If enhanced vigilance to detect malaria cases is not maintained by healthcare providers, the threat of malaria re-establishing in the country is high.

Given its diversity and reach, the private health sector is an important partner for malaria surveillance. The only information currently available from the private sector on the role of the private sector in malaria surveillance is the number of cases detected. The percentage of malaria cases identified in the private sector has increased over the past few years (10/47 in 2014, and 12/36 in 2015). The purpose of the study was to determine the number of diagnostic tests carried out, the number of positive cases identified and the referral system for diagnosis in the private sector and to estimate the costs involved. This information can be used for evidence-based decision making to harness the human and financial resources of the private sector healthcare providers to keep Sri Lanka “malaria-free”.

## Methods

### Sample selection

This descriptive prospective study was carried out in registered private hospitals and laboratories within the Colombo District of the Western Province of Sri Lanka. This district was selected for several reasons: (a) it is the most populous district of Sri Lanka in which the commercial as well as administrative capitals of Sri Lanka are located [5], (b) it has the highest number of registered private healthcare providers in Sri Lanka [6] and (c) the number of reported imported malaria cases in Western Province, a traditionally non-endemic area for malaria, has increased over the past few years (2013: 63/95, 68.4 %; 2014: 34/49, 69.4 %; 2015: 26/36; 72.2 %) (Personal communication, Director, National Anti Malaria Campaign, Sri Lanka, 2016). In 2015, the highest number of malaria positives, all imported malaria cases, was reported from the Colombo District (24/36, 66 %). Most tourists visiting the country (and locals returning from abroad) would enter or pass through Colombo as the main international airport in Sri Lanka is located 40 km from Colombo. The Colombo District also has the largest private hospitals in the country, each with a capacity of over 100 beds, thus attracting those seeking healthcare from other parts of the country as well as tourists and foreign visitors.

Malaria diagnostic services by the private sector in the Colombo District are provided by (a) hospitals (with clinic and in-patient facilities) which have their own diagnostic services and laboratories, and (b) stand-alone medical laboratories, which only provide diagnostic services.

All private sector healthcare institutions that provide diagnostic laboratory services that are registered with the regulator (Private Health Services Regulatory Council, Ministry of Health, Sri Lanka) within the Colombo District were included in the sampling frame. The investigators visited and assessed each laboratory. The non-functioning establishments (n = 46), laboratories, which do not carry out malaria diagnostic testing (n = 25) and specialty laboratories (e.g. reproductive health, eye care, n = 6) that were not likely to provide diagnostic services for malaria were excluded. Among the functioning laboratories, information was obtained on the diagnostic test use and the amount that is charged from a patient.

### Data collection

Following discussion with the management of the private sector laboratories with malaria diagnostic facilities, permission for data collection was negotiated. Data collection was done over 6 months from March to August 2015. A format for reporting on malaria diagnostic services was developed and pretested prior to the commencement of the study. Reporting cards were given to each institution and collected at the end of every month. The monthly return included the serial number, age and sex of the individual undergoing a diagnostic test for malaria (collected from the laboratory referral note) and the diagnostic test used (personal details such as name and address were not collected). The data collection form also highlighted that if a malaria case is detected, the Anti Malaria Campaign should be immediately informed. Two research assistants were recruited to follow up all laboratories during the 6-month period. Nil reporting (absence of malaria cases) was adhered to by all laboratories after reminders were sent, first by email, and then by a telephone call. Several follow up calls or visits were sometimes required to collect the information.

The investigators only collected data on malaria diagnostic tests performed and did not interfere with the management of a malaria patient or the subsequent response which was the responsibility of the respective hospital and the AMC.

For comparison of the number of malaria tests conducted with the total number of admissions and out-patient consultations in each of the hospitals, the hospital admissions registry and the out-patient department consultation registries were checked with permission from the hospital authorities.

Data were entered and analysed using SPSS statistical software (Version 20, SPSS Inc., Chicago, IL, USA). Descriptive statistics were summarized and presented as percentages or proportions.

## Results

Two hundred and five (205) private healthcare institutions (hospitals with laboratory facilities and stand-alone laboratories) in the Colombo District were registered with the regulator (Private Health Services Regulatory Council, PHSRC). Of these, 41 laboratories were eligible to be included in the study (Table 1). Data were recorded continuously over a 6-month period from 28 laboratories and the remaining 13 laboratories failed to provide information in spite of repeated reminders and visits (response rate: 68.3 %). The 28 laboratories from which data were collected included 13 laboratories in private hospitals (of which ten had performed malaria diagnostic tests during the study period) and 15 stand-alone private laboratories. Malaria diagnostic tests were performed only in five of the fifteen stand-alone private laboratories, which included three centres that screen for malaria for foreign employment. Malaria screening tests for foreign employment were counted separately. The remaining 10 stand-alone laboratories did not receive any requests for diagnosis of malaria by microscopy or RDT during the study period.

**Table 1:** Description of private sector laboratories in Colombo District

Laboratory	Number (%)
Total Laboratories registered in the Colombo District	205 (100.0)
Specialized Units	6 (2.3)
Laboratories referring blood smears to larger centres	72 (35.1)
Labs which do not do testing for malaria	25 (12.2)
Closed Laboratories	46 (22.4)
Laboratories meeting inclusion criteria	41 (20)
Labs which sent data continuously	28
Private hospitals	13
Private stand alone laboratories	12
Other Laboratories	3
Labs which failed to send information	13

The breakdown of the number of malaria diagnostic tests carried out during the study is given in Table 2. Of the individuals who were investigated for malaria in the private sector laboratories (n = 2046 by microscopy and 88 by RDTs), 951 (44.6 %) were tested due to requests from doctors based on clinical suspicion (863 by microscopy and 88 by RDTs); 8 (0.37 %) individuals got themselves tested by microscopy following foreign travel. The rest of the samples tested for malaria were routine testing at blood banks of these hospitals (n = 1175, 55.1 %). 14 blood smears were examined for malaria at the two stand-alone laboratories, which were all based on requests from General Practitioners (total malaria diagnostic tests based on doctors requests and self testing = 973). Eight malaria positives were reported from four hospital laboratories during the 6-month period and one malaria case was detected from one of the two stand-alone laboratories. All positive cases (n = 9) were notified to the Anti Malaria Campaign within 24 h of diagnosis. The diagnoses of malaria made in the private sector laboratories were confirmed as 100 % accurate on re-testing of the patient by the AMC using microscopy and RDT. The three private sector stand-alone laboratories involved in medical fitness assessment for foreign employment examined 12,813 blood smears for malaria parasites. None of blood smears were positive for malaria.

**Table 2:** Number of diagnostic tests carried out for malaria from March to August 2015 in private sector institutions

Private sector institutions (n-15)	Diagnostic method	
	Microscopy	Rapid diagnostic testing (RDT)
Hospitals (n-10)	2046	88
Stand alone diagnostic laboratories (n-2)	14	
Other laboratories (n-3)^a^	12,813	
Total	14,873	88

^a^Accredited laboratories conducting malaria diagnostics as a part of routine medical examinations for employment and travel purposes

Of the patients diagnosed with malaria during the study (n-9), five were Sri Lankans who had acquired the disease while travelling overseas. Others were foreign national, two each from India and Pakistan. In the private hospitals, five patients were diagnosed when admitted with fever and three were diagnosed at out-patients department (OPD) consultations. Of the OPD patients, one had come for voluntary screening after foreign travel to a malaria endemic country and the others were tested because they presented with fever. The patient who was diagnosed at the stand-alone private laboratory was an Indian national who was referred for testing by a General Practitioner when he presented with fever.

During the 6 month study period, the total number of patients seen in the OPDs (n = 295,325), the number of patients admitted to the medical wards (n = 29,515) and number of patients admitted to paediatric wards (n = 6976) in the ten private hospitals included in the study was 331,816. The number of patients tested for malaria was 959 (0.3 %).

Rapid diagnostic tests were only available in hospitals. The patient cost for a RDT test in the five private hospitals varied from 8.5 USD to 13.8 USD per test. The average cost was 12.1 USD. The average inpatient cost of microscopic examining of a blood smear for malaria parasites in the private sector was 2.5 USD; the cost of microscopy was cheaper by approximately 0.35 USD when carried out in the OPD. All laboratories used the Leishman method of staining of thick and thin blood smears.

Of the 2148 requests for malaria testing by the private hospital laboratories and stand-alone laboratories, basic details such as age and sex were indicated in approximately half of them (n = 1160) (Table 3).

**Table 3:** Age and sex distribution of persons in whom malaria diagnosis was requested (n = 2148)

Characteristic	Number (%)
Age	
<12	29 (2.5)
13–20	125 (10.7)
21–30	459 (39.6)
31–40	271 (23.3)
41–50	161 (13.9)
51–60	78 (6.7)
>60	37 (3.9)
Gender	
Male	973 (82.7)
Female	201 (17.2)

Technical staff working at the private hospitals’ laboratories covered in this study included qualified Medical Laboratory Technicians (MLT) who had completed a comprehensive course which includes a detailed training on malaria diagnosis as well. It was not possible to find out the qualifications of the technical personnel who carry out malaria diagnosis in all stand-alone laboratories (as the owners of the laboratories were not keen to divulge this information) but 10 out of the 12 laboratories had a trained MLT, including the two laboratories which got requests.

Out of 36 malaria cases reported in Sri Lanka in 2015 (all imported cases), twelve each were diagnosed in the private sector and government hospitals in the Colombo District (66 % of the cases in the country). Of the rest, two were diagnosed at government hospitals in the other two districts of the Western Province (Gampaha and Kalutara districts), and ten from the government hospitals in other parts of the country (Personal communication, Director, AMC).

## Discussion

Sri Lanka has been free from indigenous malaria transmission since October 2012. Imported malaria infections continue to be reported in the country and steps are being taken to mitigate the risk of re-introduction of malaria [7]. A robust and responsive surveillance system is critical for prevention of re-introduction of malaria as Sri Lankan nationals travelling overseas and foreigners arriving from malaria endemic countries are the key groups in whom imported malaria has been reported. Enhanced vigilance in patients presenting with fever with a travel history to a malaria endemic country by clinicians, and early diagnosis and treatment of malaria are critical, as delays or failure to diagnose imported malaria infections may considerably increase the risk for re-establishing local transmission [7, 8].

As in other countries, the health care needs of Sri Lankans are served by both the public and the private sectors in parallel. Government health care services are offered free of charge to its citizens but its institutions are most often overcrowded specially in metropolitan areas such as Colombo. There are private healthcare providers that offer the same services in less congested surroundings which middle class Sri Lankans can afford. Foreigners, especially tourists, preferentially seek treatment from the private health sector. A similar practice is observed for the foreign workforce in the country (mainly Indian and Chinese nationals) providing labour to industrial, construction and agricultural projects. In general, most people who travel overseas, and therefore at risk of contracting malaria, are those who can afford and prefer to access the private healthcare system when sick. It is estimated that the private sector contributes to 50 % of the total annual outpatient hospital consultations in Sri Lanka [9]. This, and the fact that one half of all malaria cases reported from the Colombo District (12/24) and one-third of all malaria infections reported in 2015 were from the private sector (12/36), points to an extremely important role that the private health care institutions play in malaria surveillance and notification. There is no published literature on the contribution of the private health care sector to malaria surveillance in Sri Lanka.

Colombo, the commercial Capital of Sri Lanka, is not only the most populous district in Sri Lanka (11.9 % of Sri Lanka’s population) with the highest population density but also has the highest number of registered private healthcare institutions and laboratories. Colombo is traditionally a non-endemic district for malaria. Entomological surveillance activities carried out routinely by the Anti Malaria Campaign have not found primary vectors of malaria in Colombo for decades. With the interruption of indigenous transmission, the relative proportion of imported malaria cases detected in Colombo and the Western Province has increased. Therefore, it is important to address physician complacency and awareness of malaria in both government and private sector healthcare institutions in the country especially in Colombo. In 2012, in nearly 50 % of indigenous cases and in 50 % of imported cases, more than 5 days had elapsed from the onset of fever to referral for a diagnostic test for malaria. Despite the widespread availability of diagnostic services, there was a delay of more than 10 days from onset of fever to investigation in 26 % of locally acquired infections and 18 % of imported malaria cases [4]. In 2013, 63 % of imported malaria cases were diagnosed with malaria after more than 5 days of the onset of fever and nearly one-third was diagnosed after more than 10 days of the onset of fever. In some cases more than a month had elapsed before the patient was investigated for malaria [4].

In order to improve patient referral for malaria diagnosis, several remedial measures are in place. One is educating doctors and laboratory staff regarding the need for testing for malaria in patients who are likely to have the disease according to the guidelines issued by the National Anti Malaria Campaign [10]. All healthcare institutions in the country including the private sector hospitals have received the current guidelines for testing for malaria as a government circular issued by the Ministry of Health, Sri Lanka [10]. Further, regular in-service training programmes of the Anti Malaria Campaign for the laboratory staff involved in diagnosis of malaria have targeted not only those who are in the state sector but those in the private sector as well.

However, as requests for laboratory investigations are made at the discretion of the treating physician, a direct one-on-one awareness raising cum advocacy programme may be more effective in getting guidelines into practice. A caveat to additional testing is the direct cost to the patient. Unlike in the government sector, in the private sector the patient has to pay for all investigations and doctors tend to be restrictive to the absolutely necessary investigations based on their differential diagnosis to minimize the out-of-pocket expenditure to the patient. In a situation as exists in the country today, where malaria has been eliminated and there is absence of evidence of any indigenous transmission for consecutive years, the priority of malaria in a differential diagnosis list for an average patient with fever is low unless there are specific risk factors. This is where taking a travel history becomes critical. A recent history of travel to a malaria endemic country in a patient presenting with fever should never be ignored and referral for a malaria diagnostic test should be a must.

Only two stand-alone laboratories got requests for malaria testing, all from general practitioners who probably do not have their own laboratory services and there by have no other choice but to send their patients to a private sector laboratory. Overcrowded government hospitals also send their requests to private laboratories, many which have sprung up close to major government hospitals as the tests can be done more quickly but no referrals from government hospitals to private sector laboratories were observed in this study. The continuing medical education programmes conducted by the AMC should target general practitioners and emphasize on malaria awareness and expanding the coverage and frequency of testing for malaria.

Despite the low referral rate for malaria diagnosis, Sri Lanka has been maintaining an Annual Blood Examination Rate of about 5 % during the last 5 years based on data provided by government health care institutions. The denominator of the ABER is the Sri Lankan population and technically not the at risk population. With malaria elimination, it is not possible to define the at risk population. The ABER is maintained at 5 % through passive case detection, active case detection and routine testing of donor blood samples to blood banks throughout the country.

With the rapid expansion of non-state run laboratories in Sri Lanka, it is now mandatory that all these institutions be registered with the regulatory authority under the auspices of the Ministry of Health. As in the other developing countries, ascertaining the quality of malaria testing in the private laboratories is an enormous challenge. An accreditation system for malaria diagnosis including the engagement of the private sector is currently being developed by the AMC. The AMC also provides in service training free of charge to MLTs from the private sector; due to most service agreements being contract based, it is not sure how many of these trained personnel will remain in active service. Though microscopy is the gold standard and cheaper option for diagnosing malaria, RDTs offer a quick and easy way of making a diagnosis on the spot and may be more user-friendly for doctors, especially for general practitioners. A previous study, conducted in government institutions when malaria was endemic and when RDTs had just appeared in the market, reported that the costs of performing a RDT and per case detected were approximately 14 and 20 times more than those of blood smear examination, respectively [11]. The difference in the prices in the private sector in this study is much less. As there is a price variation among the RDT kits available in different private hospitals, the possibility of regulating the price through a government assisted mechanism for the import of quality assured RDTs may be considered as an investment in sustaining the malaria free status in Sri Lanka by prevention of reintroduction.

AMC works in close liaison with the private sector. There is a dedicated focal point with a 24-h hotline to which any suspected or confirmed malaria case can be reported. Surveillance staff at the AMC headquarters has initiated a strong rapport with the private sector. AMC provides diagnostic services for immediate confirmation and provides free treatment as per national treatment guidelines to all malaria patients in the private sector. Essential anti malarial drugs such as artemisinin combination therapy and drugs for the treatment of severe malaria are only available with the AMC as the availability and sale of these drugs in the market is controlled. This not only ensures that WHO pre-qualified drugs are used to treat malaria in Sri Lanka, but also warrants that all diagnosed cases are notified to the AMC to obtain treatment. Real time reporting of malaria including nil-reporting from the private sector is now being initiated by the AMC using the same formats used for this study. Engaging the private health sector in a routine malaria surveillance system is a challenge encountered by the national programmes that have embarked on malaria elimination. Sri Lanka, having eliminated the disease since October 2012, has been making progress in engaging the private health care facilities for further strengthening of malaria surveillance.

## Limitations

This study was restricted to one district in the country. However, this district is the most populous in Sri Lanka that has the largest number of private health care institutions and reported the highest number of imported malaria cases in Sri Lanka since interruption of locally transmitted disease. The findings may have been different if other districts were also included. The overall response rate of eligible private sector laboratories was 68 %. Even though the response rate was less than what we expected, the laboratories of all the bigger private sector hospitals in the district, which are most likely to be visited by malaria patients, participated in the study. Repeated reminders and visits to hospitals and laboratories by the research team would probably have contributed to the response rate. Formal arrangements will have to be made to obtain regular feedback from private health care providers if the AMC engages them in the malaria surveillance system. The reasons for testing (or not testing) patients for malaria were not investigated from a physician’s point of view.

## Conclusions

Private sector hospitals and laboratories in the Colombo District have diagnosed one-third of all malaria cases reported nationwide in Sri Lanka in 2015. Engagement of the private sector in malaria surveillance will be critical for an effective national surveillance system for capturing of all malaria cases and timely treatment and response. Quality assurance of private sector malaria diagnostic services and reporting practices would be vital for prevention of re-introduction of malaria to Sri Lanka.

## Abbreviations

AMC: anti malaria campaign
USD: US dollars
MLT: Medical Laboratory Technicians
OPD: Outpatients Department
PHSRC: Private Health Services Regulatory Council (PHSRC), Ministry of Health, Sri Lanka
RDT: rapid diagnostic test
WHO: World Health Organization
